# Impacts of COVID-19 on African Migrants’ Wellbeing, and Their Coping Strategies in Urban and Regional New South Wales, Australia: a Qualitative Study

**DOI:** 10.1007/s40615-023-01806-z

**Published:** 2023-09-28

**Authors:** Peter Bai James, Kathomi Gatwiri, Lillian Mwanri, Jon Wardle

**Affiliations:** 1 National Centre for Naturopathic Medicine, Faculty of Health, Southern Cross University, Lismore, NSW 2480 Australia; 2 Faculty of Pharmaceutical Sciences, College of Medicine and Allied Health Sciences, University of Sierra Leone, Freetown, Sierra Leone; 3 Centre for Children & Young People, Faculty of Health, Southern Cross University, Gold Coast, Australia; 4 Centre for Public Health, Equity and Human Flourishing, Torrens University Australia, Adelaide, SA 5000 Australia

**Keywords:** COVID-19, African migrants, Impact, Coping mechanisms, Australia

## Abstract

**Aim:**

As the COVID-19 pandemic response continues to evolve, the need to protect more vulnerable populations in society becomes more apparent. Studies are still emerging on how different population groups have been impacted by the COVID-19 pandemic. Our study explored the impact of COVID-19 for African migrants in New South Wales, Australia, and their coping strategies.

**Methods:**

We employed inductive, exploratory qualitative interpretive research design using individual semi-structured in-depth interviews with 21 African migrants.

**Results:**

COVID-19 lockdowns disrupted the African sense of community. Social isolation, financial insecurity due to joblessness, or reduced working hours led to stress, frustration, anxiety, sadness, loneliness, and depression. On the other hand, COVID-19 lockdowns allowed for more family time, reflecting, and appreciating the gift of life and personal intellectual growth. Despite such challenges, there was much community support, especially from religious organisations. Support from government agencies was available, but access was hampered by misinformation, digital literacy, and immigration status. Holding on to religion and faith was a key coping mechanism, followed by indulging in self-care practices such as healthy eating, exercise, Yoga, meditation, sleep, and limited interaction with social media.

**Conclusion:**

The COVID-19 lockdown disrupted the collectivist culture of African migrants and had untoward socioeconomic impacts that affected their wellbeing, many of which reflect an exacerbation of pre-existing inequities. To ensure that African migrant COVID-19–related health and wellbeing needs are met, the African migrant community must be actively involved in every facet of the NSW COVID-19 and other future outbreak response efforts.

## Background

In Australia, migrants were identified as one of the at-risk groups for COVID-19 response policy formulation and implementation [1, 2]. While the impact of COVID-19 on migrants may vary from one migrant group to the other based on culture or ethnicity, signalling the need for accessible and culturally appropriate COVID-19 response interventions, the scholarship on the impact of COVID-19 on the health and wellbeing of the general migrant population in Australia and African migrants, in particular, is limited.

In Australia, the Government’s approach in limiting the spread of the coronavirus (SARS-COV-2) via its public health measures, had a significant economic and psychosocial impact on the general population. The enforced restrictions forced many non-essential businesses and activities to close, causing many people to lose their jobs and livelihoods. Restrictions on social gatherings meant that families and loved ones were unable to spend time together. Ceremonial services which often bring communities together and are seen to foster connections that bolster wellbeing such as weddings, birthdays, and engagements were halted. Transition ceremonies such as memorial or burial services in situations where a loved one passed on were made inaccessible [3, 4]. In healthcare settings, patient access certain medical procedures deemed as non-urgent were suspended. Interactions between patients and healthcare workers were forced to adopt a different service delivery model, such as telehealth [5, 6].

There are already considerable well-documented challenges affecting migrants in Australia that amplify their vulnerabilities [7, 8]. Additional stressors that the COVID-19 pandemic presented were predicted to worsen existing health, psychosocial, and socioeconomic challenges or create new ones, further intensifying existing structural inequalities. A recent UK study reported that the use of telehealth services had excluded migrants from accessing health services due to lack of digital literacy, access to technology, and language difficulties [9]. The study further reports that migrants hold views about COVID-19 and its vaccines, ranging from acceptance to mistrust [9]. Another study on Nepalese migrants found that issues around unstable employment conditions, perceived lack of social support, obligation to send money home, and difficulty in accessing services due to the language barrier have adversely impacted their health and wellbeing. A scoping review on temporary migrant workers and international student in Australia, Canada, and New Zealand reported that these groups were excluded from accessing health services and social protection during COVID-19, and that exploitation, precarity, and racism were key factors responsible for their exclusion [10].

Some studies have reported challenges that emerge during infectious disease outbreaks resulting in increased racialised harassment, hate speech, and physical and emotional abuse towards migrants, especially from Asia and Africa. For example, during the Ebola pandemic African migrants globally were subjected to racial discrimination, and limited access to access health services [11, 12]. A study in South East Queensland showed that in Australia, there were increased racial moral panics about African migrants during the Ebola outbreak in West Africa [13]. Similar experiences were reported by Asians during the SARs epidemic [14]. In 2020, political narratives about COVID-19 being a “China-virus” increased anti-Asian racism [15]. Such racial panics often impact trust towards professionals and hinder desire to utilise government services such as awareness and prevention activities, screening, testing, treatment, and vaccination [16]. In addition to these barriers, previous negative experiences interacting with the healthcare system may foster mistrust and undermine preventative measures to contain infections and spread among migrant communities [17, 18].

## COVID-19 and African Migrants in Australia

The African migrant population in Australia is growing, and it is estimated to be 345,000, of which many are refugees [19, 20]. Many post-migration factors, such as cultural shock, language barriers, breakdown of family ties, social isolation, and discrimination, have impacted African migrants’ wellbeing and influenced their access to healthcare services [21, 22]. Some of these stressors are disproportionately common among African migrants. For example, an Australian study reported that Black-African migrants are more likely to face discrimination than other non-Black migrant groups [23]. Also, the 2010 Australian Human Rights Commission’s report for Africans on social inclusion and health discovered high mental health issues, inadequate health care, and low access to mental health services among African migrants in Australia [24]. The decision by state authorities to deploy the police and the military to enforced COVID-19 rules in Sydney suburbs highly populated by migrants was considered discriminatory as such actions were not implemented in non-migrants dominated local government areas in Sydney [25, 26]. Such actions led to increased lack of trust towards government initiatives.

Outside of the COVID-19 context, studies have shown that African migrants have various coping mechanisms to help them deal with the structural and interpersonal stressors of migranthood [27]. These coping strategies include access to social support from their family, friends and community, religion, willingness, and capacity to work and succeed [28, 29]. For the most part, African migrants could not use these coping mechanisms during COVID-19 following the public health restrictions that prevented people from visiting friends and loved ones or practising their faith in a communal setting [28, 29]. As a collectivist culture, Africans reported increased vulnerability during COVID-19 due to a sudden lack of community connections [28, 29]. Such disparity is likely to be more profound for those whose values are more socially geared.

The limited empirical evidence on African migrants’ health and wellbeing during the COVID-19 pandemic maybe due to lack of research funding [30] or limited Afrocentric understandings of how different African communities deal with or cope with traumatic experiences within their family settings. Another challenge is that some studies tend to homogenise migrant groups therefore, complicating any efforts for nuance and heterogeneous experiences [31–33]. Monolithic perception of migrants as a single entity may have unfortunate policy and practice implications due to the failure to address the unique challenges that particular migrant groups face.

Given the above context, there is a growing need to synthesise evidence that will inform COVID-19–related policies and interventions targeting African migrant communities to improve their wellbeing and reduce the socioeconomic burden associated with COVID-19. This study intends to contribute to filling the nascent knowledge gap in the COVID-19 scholarship as it relates to African migrants in Australia by exploring the impact of COVID-19 on their wellbeing in New South Wales. Specifically, this study will explore African migrants’ health and wellbeing, support structures, and coping strategies in urban and regional New South Wales.

## Methods

### Study Design, Setting, and Population

We employed inductive, exploratory qualitative interpretive research design using individual semi-structured in-depth interviews, given that there was less information on the topic of interest and our study data will be based on the lived experiences of respondents. A phenomenological approach [34] and an Afrocentric construct [35] underpinned the theoretical basis of our study. We conducted our study in urban and regional New South Wales (NSW). Adults (≥ 18 years), male and female African migrants (skilled migrants, refugees, asylum seekers, and students on temporal visas) who speak English and reside in urban and regional NSW were invited to participate in the study. We recruited this group because African migrants in Australia are mainly composed of skilled migrants, refugees, and asylum seekers and international students on temporal visas. Participants were recruited using purposive sampling via snowballing technique. Recruitment flyers will be distributed through known networks and migrant support groups, charities providing healthcare-related support to African migrants, social media networks of African migrants in NSW, and the social contacts of the study investigators. We recruited and interviewed 21 African migrants (skilled migrants, refugees, and asylum seekers) as data saturation was achieved [36]. We achieved data saturation in the last few interviews as we began to hear similar stories and narratives that did not provide unique or new nuanced experiences in the discussions provided.

### Data Collection

The principal investigator (author 1) conducted a semi-structured, in-depth interview using an interview guide (see attached) via telephone or online through Zoom. Author 1 is a Sierra Leonean skilled migrant with a PhD in public health. He is trained in qualitative research and has previous experience conducting semi-structured interviews among vulnerable populations such as Ebola survivors. In a situation where the principal investigator is knows by the participant, another member of the research team who is a skilled African migrant (author 2) conducted the interview. The available literature informed the design of the interview guide on the impact of COVID-19 on migrant wellbeing and coping strategies [9, 37–39]. Interviews were conducted in English at an agreed time, and they lasted for 40–60 min. Author 1 took field notes and maintained a personal reflective journal throughout the study. The principal investigator recorded his own experiences, views, feelings, and biases as he worked with African migrants and their experiences and views. This journal was used as a catalyst for discussion and shared reflection with the research team.

The journal served as an effective tool for reflectivity where assumptions, beliefs, and attitudes were recognised and understood in the context of the study. The journal does not claim to erase these beliefs, but it renders them visible to the analysis of the study and enhances the credibility of the study as it serves as an audit of the research process. Documenting these assumptions, beliefs, and attitudes and openly sharing them with the rest of the team functioned as a form of trustworthiness and accountability to the way we contextualised and interpreted participants’ view and experiences during data analysis. As an ‘insider’ researcher, the principal investigator utilised his own lived expertise to build rapport and develop trust with participants. Various studies have shown that research conducted by members outside of the marginalised and minoritised frameworks can hinder truthful story telling due the colonial legacies of being misrepresented in dominant research frameworks [40–42]. This insiderness while still situated operated in a dynamic where the researcher has significant power, played a significant part in diminishing the power dynamics between researchers and participants and allowed participants to freely express themselves without fear of judgment or reprisal.

A $50 gift card was given to each participant as a sign of appreciation for their time and energy to participate in the study. The gift cards were given to those who met the study’s inclusion criteria and completed the interview.

### Data Analysis

We employed a qualitative framework approach to analyse our data. The framework approach allows the researcher to systematically label, classify, organise, and interpret the data while ensuring rigour, transparency and robustness to the analytic process [43, 44]. The framework approach has been shown to be reliable in healthcare research [44, 45] and does not align with any epistemological, philosophical or theoretical framework [44]. A reputable transcription company transcribed the audio files. We ensured that a confidentiality agreement was in place with the transcribing company to protect participant confidentiality. To ensure rigour, author 1 read the field notes and translated transcripts while listening to the audio recordings of each interview session to verify data sources and ensure the accuracy of the transcription process. The transcript of the interview done with each participant was shared with that participant to confirm whether the meaning of what he/she said during the interview was correctly captured. After confirmation with study participants, the non-identified raw data (transcripts) were shared with members of the research team (first author and co-authors) and imported into N-Vivo version 11 software where all authors read the transcripts, retrieved various portions of the data, developed initial codes, which were discussed among authors. The principal investigator grouped the agreed codes to form framework categories and tested the framework categories with two transcripts, and the outcomes were discussed with co-authors. This stage underwent several iterations to develop clearly defined framework categories, which were applied to the remaining transcripts [43, 44]. Summarised portions of interest in the transcript texts were entered into their relevant categories and linked with the original transcript text for each participant. The charting process led us to identify patterns and established themes from the data to get an overall impression of African migrants’ experiences during the COVID-19 pandemic and their coping strategies. We chose quotes to represent key themes based on their quality and representativeness. The study findings were presented in line with the consolidated criteria for reporting qualitative research guidelines [46].

### Ensuring the Trustworthiness of the Study

The trustworthiness of this qualitative study was ensured through credibility, confirmability, transferability, and dependability [47]. To ensure credibility, the principal investigator kept a reflective journal, enabling a detailed record of relationships and personal reflections. Also, we purposively sampled African migrants to ensure we get participants with diverse sociocultural backgrounds, migration status and varied experiences. Purposive sampling allows for the selection of information-rich cases, which creates an opportunity for new ideas to be included that are relevant to the topic of enquiry [48]. Confirmability was ensured using ‘participants’ quotes to explain how the data was interpreted. Direct quotes from the transcripts were used to illustrate the main themes. These quotes were validated through participant’s feedback, an audit trail of the data collection and analysis process, and researcher triangulation to ensure a valid interpretation of the African ‘migrants’ views and experiences. Transferability was addressed by providing a detailed description of our study background, method, and findings. Dependability was achieved by ensuring that rich data grounded our analysis. Audit trails of the data collection and analysis process, such as audio files, transcripts, and field notes, were maintained and routinely consulted during data analysis and interpretation. Also, all transcripts were entered into NVivo software, where all authors read, familiarised themselves, developed individual codes, discussed, and agreed on key emerging themes and quotes.

### Ethical Consideration

Ethics approval to conduct the study was obtained from the Human Research Ethics Committee of Southern Cross University (Approval Number: 2021/128). Each recruited participant was sent the patient information sheet, which explains the purpose and scope of the study and the option of opting out if they felt uncomfortable during the interview. Both verbal and written consent was sought from each respondent. Participant information sheet and consent form were sent to each potential participant prior to the conduct of the interview. Signing the consent form was taken as an expression of their willingness to participate, which was confirmed by verbal consent before the start of the interview.

## Results

Table 1 summarises the sociodemographic characteristics of African migrants that participated in the study. Close to half of them were between the ages of 30–39 years (*n* = 9; 42.9%) and females (*n* = 10; 47.6%). Close to two-thirds were Australian citizens (*n* = 13; 61.9%) and half of them were originally from Nigeria (*n* = 4; 19.0%), Sierra Leone (*n* = 4; 19.0%), and South Sudan (*n* = 3; 14.2%). One-third resided in regional New South Wales (*n* = 7, 33.3%) The following themes emerged from our data analysis: impacts, support systems, and coping mechanisms.

**Table 1:** Sociodemographic characteristics of African migrants (*N* = 21)

Characteristics	Variable	*n* (%) mean (SD)
Age group (years)	< 20	1 (4.8)
	20–29	4 (19.0)
	30–39	9 (42.9)
	40–49	2 (9.5)
	50–59	4 (19.0)
	≥ 60	1 (4.8)
Gender	Male	11 (52.4)
	Female	10 (47.6)
Immigration status	Citizen	13 (61.9)
	Permanent resident	3 (14.3)
	Temporal resident	4 (19.0)
	Bridging visa	1 (4.8)
Length of time in Australia	Mean (SD)	10.19 (± 6.65)
Occupation	Employed	17 (81.0)
	Unemployed	4 (19.0)
Educational level	Tertiary education	20 (95.2)
	High school	1 (4.8)
Country of origin	Burundi	1 (4.8)
	Demographic Republic of Congo	1 (4.8)
	Ethiopia	1 (4.8)
	Ghana	1 (4.8)
	Kenya	1 (4.8)
	Nigeria	4 (19.0)
	Sierra Leone	4 (19.0)
	South Sudan	3 (14.3)
	Tanzania	1 (4.8)
	The Gambia	1 (4.8)
	Uganda	2 (9.5)
	Zimbabwe	1 (4.8)
Residence	Regional New South Wales	7 (33.3)
	Metropolitan New South Wales	14 (66.7)

## Psychological, Economic, and Social Impacts of COVID-19

### Mental/Psychological Impact

COVID-19 lockdown created huge anxiety among African migrants. Sources of anxiety were about uncertainty of not knowing what was going to happen, given the evolving public health responses to the changing dynamics of the SAR-COV-2 virus, listening to news on TV about COVID, fear of being infected with the SAR-COV-2 virus and spreading it to loved ones, worrying about being unemployed or laid off, running out of basic households’ items and concern about the health of loved ones back home in Africa.

“I know as a big person, if I get the COVID, you know, I can cope with it, but where am I going to be able to separate with my little ones, you know? If I get it, obviously, they will get it, and dealing with that becomes very hard and you actually start worrying about the children; what if they get COVID? What is going to happen to them? You know, would they be well or not? You know. All that anxiety about thinking what it is like, what is going to happen, all of this. Yeah, it’s a bit too much.”

“I was worried about not knowing what going to happen next. Also, Uh, it’s like my thoughts are in all places. I can’t concentrate. I was currently overthinking and overstressing about how to do certain things”.

In addition to feeling anxious, some participants also stated that they felt depressed due to being isolated, lonely, unable to visit friends or being visited. For some, depression symptoms manifested through inability to do daily chores, getting out of bed, oversleeping or under sleeping, being easily irritable, or overly frustrated about every aspect of their lives. As one participant puts it.

“Um, my ability to do the things that I used to do then, I can no longer do them now. I-I feel like getting out of bed is-is so difficult. I slept a lot.”

Another participant added that being away from their family, their children and being alone during the pandemic affected their mental health.

“I was not happy at all. You know, I was living a sad life because I was alone. I don’t have anyone, uh, living with me. I live alone and my children are living far away. So, uh, it came to a time, uh, I-- it disturbs my sleep anyway. I could not sleep as normal as I used to. My sleeping pattern changed completely. Sometimes I wake up in the night and I cannot sleep because I was thinking too much.”

For some, the increased vulnerability in their mental health state also affected their physical health as well.

“Before the pandemic, I was active. I used to go for an hour or two hours walk in the park with some of my African friends. We discontinued that, um, due to the pandemic and, um, as a- as a result, I became lazy, you know, in taking care of myself and all that. And, um, yeah, I started eating a lot and putting on weight and, of course, when you are putting on weight, a lot of things will be, um, affected as well.”

The covid-19 lockdown means they were unable to hang out with friends as they used to prior to the COVID-19 outbreak. Being stuck at home all the time made them feel sad and moody.

“Oh, look, the-the-the thing is, um, with the kind of job that I am doing, like sometimes you need to come home and take a breather. But with, um, it’s-- It-it becomes hard when you come home, and you can’t do anything else. Like, you-you’re stuck in one place. Um, you can’t- you can’t like, um, go out to take, um, like take a drink with friends or other things. I started feeling, um, like you have this moody kind of feeling. You know, you-- Uh, uh, sometimes I sit down, and I start crying because, um, like I don’t feel good about myself you know?”

### Social Impact

COVID-19 lockdowns disrupted the African sense of community. For most African migrants, living alone without physical interaction with friends and family members is a contradiction to the collectivist part of African culture. The loss of social connection and belonging was more profoundly experienced for those whose families were not with them-Families for African migrants in Australia may look like fellow Africans or country mates who have formed connections after arriving in Australia. Countrymates may consider each other families especially when their biological families are in Africa.

“I used to go to different places with them, but because of COVID, I cannot go anywhere. I was supposed to be-- Just stay at home. So, it was really a different experience for me and, um, a bit stressing. Like I said, in back in our country, we have a social life, so we cannot live-- It’s like we cannot live without other people around us.” Or unable to physically interact with acquaintances.”

Another participant put it this way.

“I’m losing- I’m losing my mind because I-I can’t see the people I need to see. We used to go for-for lunch whenever we got a space, me-me and my friends, and that is no more. We used to have activities that we used to-to engage in whereby we could come, eat, discuss, talk, enjoy. That has gone. That is no more. Our support that we used to have that comes from, uh, uh, the strength of our culture has gone.”

For some African migrants, COVID19 restrictions imposed by the NSW government led to them not being able to attend key family functions, which affected their close relationships, and opportunities to connect.

“It has just taken people backward and-- or progress. Since it starts in 2019, I haven’t travelled out of this country. I haven’t gone anywhere. I have lost opportunities. I have lost a relationship because of this because I was in a distance relationship. I’ve lost that one because, um, I can’t travel and all of that, and--distance, you know, breaks relationships, so all of those things. I’ve missed my sisters’ weddings. Like two of them I’ve-- I missed-- I missed that one in 2020 and the other in 2021”

### Financial Impact

COVID restrictions also affected participants work patterns and invariably their finances. A handful of them lost their jobs, and for some, the number of working hours were reduced, especially those on casual employment. This meant a reduction in income, which affected their ability to take care of family expenses and sending money to their relatives in their home countries.

“Uh, I used to be a lecturer, but during COVID, I lost my job. I had to resort to many "odd jobs to make ends meet. My wife also used to manage a store somewhere, but because of COVID, the store ran out of business and was laid off.”

Another participant stated;

“Yeah, I used to send them [family back home] money, but 'because of COVID-19, I had to reduce the amount I used to send to them as I didn’t have a- lot of money. Also, it was tough to paying my bills and put food on the table for my family”

Due to the financial stress many, participants stated that they were allowed to access their superannuation as a result of the federal government policy that allowed people who were financially impacted by COVID-19 to offset the economic hardship they were experiencing. Others reflected that they had to be frugal and cut down on the few social activities that they were allowed to participate in the midst of COVID restrictions.

“You know, I have to be, hey, be strict on how I spend my money. Then you start, you-you cancel some of these expenses. You cancel, for example, going to the gym, you cancel going to the swimming pool, for example. You-you-you cancel going out, you know, uh, you cancel all these stuffs, which in a sense could maybe be like kind of coping strategies with,”

Some participants who work in the social service sector, especially aged care and disability services, spoke about how they their work dynamics completely disrupted. They were mandated to adopt barrier care to protect themselves and their clients. This includes compulsory use of gloves, face mask, personal protective equipment and adhering to 1.5 m physical distance. In addition to their own anxiety about their own health, these requirements, though important, constituted additional labour which accumulated to extra physical and emotional toll that invariably affected work efficiency.

“It was too hard to work in such an environment-- You have to be extra careful. …- because it was a new thing, so you had put on gloves and your mask. It was just difficult, you know? it was something new, so it was really affecting, uh, -our work per se, you know, the efficiency and the delivery of the care. you need to get it right. If you don’t get it right, you’ll be in problem, you’ll either acquire COVID or you will give out COVID, you know? So that by itself makes you become ex-extra careful and being in the extra alert because you are working in the disability sector, you need to work with the client. You need to ensure that, uh, uh, he can live as normal as possible. So, the living of normal life became questionable or was redefined. So, in such case, we were working the brain and body. So, it-it affected the efficiency. So, something you’re supposed to do in two minutes, it takes you five, six minutes,”

However, some participants reported that the COVID19 lockdown did not affect their work and finances. As one stated;

“I think for me, I was quite fortunate because, throughout, you know, COVID, I did have a stable job. So, I was either working full time, while on the side, working on my business. I had a stable income coming throughout that time. And so, when things didn’t work out with my business within the period of the lockdown and COVID situation, —…-the income from my fulltime job kind of pulled me through.”

### Unanticipated Positive Impacts

Despite the highlighted negative psychological and socioecomic impacts of COVID-19, some African migrants mentioned that they were some positives of the COVID-19 on their general welbeing. For some, the COVID-19 lockdown created opportunities to spend time bonding with family and frequently checking on friends here and relatives in their home countries.

“Um, when I say positive, um, like it’s-- It has brought my, um, my family together and it also has made me to appreciate what I had with my parents. Like the kind of friendship that I have and not take it for granted. For instance, my husband worked from home and because he’s home, um, all the time, we get to spend time together when I’m home. We had to do remote learning for the kids. That actually helped us to bond more, even though it’s stressful, but we-we bonded more as a family.”

Some participants also cited that the COVID-19 lockdown allowed them to be involved in personal development programs such as improving their digital skills, or upskilling their knowledge by doing some certificate courses and online trainings from home without the burden of travelling face to face.

“I knew I cannot go out. I cannot do walks, so what I decided is, uh,[because] the Government was giving this, uh, uh, education thing. Yeah, this is the time to do further studies, upgrade[my skills during COVIDI did a graduate certificate in Disability and Inclusion. And then, now-- I’m now I’m doing psychology as well”.

Another participant stated;

“In terms of, um, professional development, you know, training, I have been now able to do a lot more also onine, which I guess if it was face-to-face, the challenge would be always, if there’s something, say, ..in Sydney, I always have to be thinking about my kids, have to find someone to look after them. So, it’s been possible for me to do a lot more.”

For some, COVID19 lockdowns allowed them to pause and self-reflect on their fast-paced lives they were living prior to COVID-19. Such self-reflection made make significant changes on the choices they made on their personal lives and careers.

“Slowing down of the pace has been- has been a positive thing because we used to be, uh, up and running every now and then. we used to work boom, boom, boom, you know? The world was-was like it was moving on a high treadmill. Right-right now, there has been some reduction in that speed. Uh, it has helped me to reflect on my life, see where have I gone wrong, and how I can improve. That has been really so crucial in my life. I think, um, what I’ve learned most is basically the importance of having a break. In most cases, we work throughout the year. Um, we always get leave, but in most cases, you may not take the leave because of what you have to do. When you take this leave, it makes it easy for you to realise how far you have achieved, and how much you have achieved, and where you heading, as compared to the other time of you’re-you’re always on-on a run and you take less time of thinking about exactly what you’re doing”

## Support Systems

### Government Support

Like other members of the Australian population, government support services were available to the African migrant community who were permanent residents and Australian citizens. Financial support for those who lost their jobs or had reduced working hours was available through a policy called “job keeper and job seeker’.

“We did get access to the COVID payment where if you got COVID and your work wasn’t paying you for sick leave and whatnot. Yeah, we got that, um, we took advantage of that one.”

Although some migrants could access and benefited from these services; for some, accessibility was hampered by huddles such as digital literacy, migration status and income level. As one participant stated; “Well, uh, for temporal resident like me, I basically didn’t get no financial support from the government”. another participant stated;

“[I got support through], Centrelink, I did. The condition that they gave me was, say, going back to work which I can’t work….. And with Centrelink, is now so frustrating. If you are not that educated in computers, they just leave you on the computer by yourself, which you can’t do. So what’s the essence of that? So all of those things is more additional frustration to you. Um, if you’re not computer literate, you can’t do anything. And they keep barging you, go back to work, go back to work. Such things like that, I wasn’t ready to take all those stress adding on to what I’m going through.”

As established, COVID-19 had a huge psychological effect on the general population, including African migrants. As such, access to psychological support was essential to preserving people’s overall wellbeing. The government extended the number of sessions that people could see a psychologist or mental health social worker on Medicare rebate. Some participants reported that they utilised psychological support available to them. One said;

“Yeah, so, I personally had to call the hotline for mental-health help. I wasn’t really in need of an urgent mental-health help, but I told them, "Look, I’m not feeling like myself. I’ve being sitting here, moving from the bedroom to the sitting room.”

Others stated that, even when it was available, they did not utilise that support. The key reason cited was that had to do with the fact that poor mental health carries significant stigma in African society and instead opted to rely on their faith and religious beliefs to get through the hard times.

“I haven’t given it a good thought. I think part of the reason, also has to do with my background. In Nigeria, you talk to God before you start talking to people. I didn’t grow up seeing people around me talking to professionals, they talk to God.”

Another participant stated;

“I got some recommendations from my supervisors to get some counselling but I never tried to get such a service. In the community where I was raised, seeking psychological support is not that common. So I think, um, we prefer to deal with it by ourselves.”

### Community Support

Support from members of the African community mainly came from close friends, acquaintances, relatives, and church members, and it mainly involved emotional support although in some instances, it was in form of financial and physical assistance such as shopping for those in isolation. It was common for people to call and send text messages, more than they used to previously, to know how their loved ones were doing and how they could help. Some participants joined online Whatsapp groups created by community members to stay connected digitally. These group chats served as a platform for people to share their concerns, check, counsel, motivate, and pray with each other. Such support was considered helpful in maintaining wellbeing during lockdown and made participants less isolated.

“When I was in isolation, members of my community sometimes would deliver food at my home. They would call, talk to, and encourage me not to worry. This was really helpful. You see, sometimes when somebody calls you and say, "How are you? How do you feel?" in itself, is good. If some people are chatting with you so you will feel happy and know that you are not alone, and that some people care about you when you are in difficulties”.

As another participant stated.

“Yeah. The African community, we’ve good aunties, and the uncles who call you every time to check on you or send you a message. How are you going, how-how’s work, how’s COVID can I help you with anything? Or I’m here if you need a hand.”

However such support was difficult to access in regional New South Wales as the African migrants in these areas are very few and sparsely distributed.

“In Armidale here, there are obviously a few Africans and they are scattered. So, there is no such big community here that I could get support from. I mostly had support from friends in Sydney and non-Africans here”.

Religious organisations, especially churches were instrumental in providing support to many of their members and the community. The support was not only spiritual and emotional, but also material support such as food hampers and help pay their bills.

“My church was also a support pillar. A support pillar in the sense that we have this, uh, food bank thing that we subscribe to and we get, uh, food from the food bank and we distribute to members and also to people around the community. The church was able to kind of still provide guidance. We weren’t able to meet face to face, but we had church services online via Zoom. My pastor made it a duty that some of us, including him, call other people just to check on them and encourage and help them deal with whatever they’re going through. And I believe this also helped a lot of families and individuals in the church including myself.”

Another participant stated that.

“Our church was atleast helping some people who were struggling financially by assisting in paying their bills, Yeah, our church has been doing that, and like just helping them with little, little bits here and there.”

## Coping Strategies

### Well-Being Practices

Many migrants indulged in self-care practices to cope with the impact of COVID-19 to maintain their health and psychological wellbeing. Some of these practices include eating healthy, exercise, Yoga, meditation, and sleep.

“Yeah, with the challenges we had during lockdown, I think we just do what we can to manage them. During lockdown, I realised that I had to focus on my well-being, which means that I had to get a routine in terms you know doing stuff like going for a walk and getting some sunshine, doing Yoga, or some sort of meditation at home. So I felt like that was a positive thing regarding the things I took on board and actively implemented during that time”

As another participants mentioned.

“In terms of self-care, I would do, yoga exercise, I’ll dance by watching YouTube video, tutorials on dance, workouts. Uh, I think those were the-, yeah, the key things.”

Some started self-medicating with home remedies and over the counter medications to prevent COVID-19.

“Yeah, I treat myself when I have flu 'cause sometimes from this flu, you don’t know if you have got COVID or not. Prevention is better than cure. I blend the garlic and hot water, mix it with lime and honey, then drink it. It is good. It’s good for flu-like symptoms and coughing.”

Others distracted themselves from the onslaught of negative COVID-19 stories by avoiding news broadcasts and instead focusing on media where they could control what they watched. These included movies and documentaries. As a participant stated;

“I would say that I really try to avoid the news, to be honest, because I feel like there’s a lot of crap.Some of the things, it makes me like more, I mean, I feel like it’s the same story every day. There’s nothing positive really on the news. And, um, I look at the headlines, too see if there an important message for me, and that’s it. So it’s like I try to limit my time on watching the news because when I hear a lot of the things, what is gonna happen, it begins to make me feel more nervous”

Religion- and faith-based wellbeing practices were critical in helping people overcome the difficulties they experienced due to COVID-19. Given the uncertainties that came with COVID-19, prayer, and faith in God offered hope that the difficult situation would soon be over. Some participants stated in particular that listening to gospel songs was therapeutic when they were feeling down or depressed.

“First of all, as a Christian, um, I just talk to God and say, "Look, even though this is a difficult time, um, I know you are with us, you are with me, you’re with my family." So, um, having that hope that God is watching over me and God is with me, um, is one of the things that I-- that has-has helped me to cope.”

“I am a pastor, a believer, a firm believer in God and the Bible. So, I have to be frank and say, yeah, that we depended on God through it all. Depending on what the promises of God told us, and we have been holding on to those, with prayers, and listening to preaching from other men of God on social media. Praying and fasting, have been helping us greatly”

### Social Media Access

Given that physical contact was not possible due to COVID-19 restrictions the use of telephone and social media platforms served as a critical means to reach out and connect with loved ones, friends, and the general community. Such platforms allowed people to express their fears, frustrations, and concerns due to COVID-19, and this was considered therapeutic for those experiencing loneliness, anxiety, and depression. Some indulged in watching movies and documentaries to distract themselves from uncertainties and anxieties associated with COVID-19. Few believed that keeping track of what was happening allowed them to make informed decisions, which helped them protect their mental wellbeing.

“Yeah, we- you know, in Africa, there is a saying, I don’t know how to translate, it is like when you-your leg is fractured or something like you-you learn how to use a stick or something like, is, we learn much on course, for example, using social media like those WhatsApp or other things, at least to-to-to try to stay a bit connected. Uh, since we lost that opportunity to meet some times where we can in a town or in a some family event or-or we-we use too much soc-social media like WhatsApp. And that is the big help because I-- as I say, the first drink of an Africa is not the beer, it-is talking”

However, some decided to limit their engagement with social media as a way to maintain their wellbeing.

“Because I was thinking too much, I have been watching, uh, a documentary film which I enjoy, documentary and sometimes, uh, I watch other programs as well on YouTube, you know, just for me to-to ease my worry”

## Discussion

This is one of the few studies that exclusively explores the impact of COVID-19 on African migrant wellbeing, support structures and their coping strategies. Our findings contribute to the growing literature on the COVID-19 impact on racially and culturally minoritised (RACM) and other marginalised communities [32, 49]. Anxiety, stress, and depression were named as the main psychological issues experienced by African migrants due to COVID-19. These psychological issues stemmed from fear of being infected with or transmitting the virus, the public health response measures to control the spread of the virus. Also, the uncertainty of not knowing what will happen, especially the evolving public health responses to the changing dynamics of the SAR-COV-2 virus was a factor. COVID-19–related socioeconomic impacts, such as reduced income due to loss of employment or reduced working hours, further contributed to mental health issues experienced by migrants in our study. Our finding supports previous studies that reported on the impact of COVID-19–related stressors on the mental health of migrant populations [31, 33, 50]. The psychological impact of COVID-19 on African migrants identified in our study will further exacerbate the existing burden of mental ill health among African migrants [51]. Thus, it is essential that the underlying COVID-19–related psychosocial stressors that led to psychological ill health should be prioritised when developing or implementing interventions. It is also vital that these interventions are socially, culturally, religiously, and linguistically appropriate to increase their uptake among African migrants. Mental health services should incorporate patients, families, and communities as active players in designing and delivering such programs [52].

Migrants are known to have unstable employment and are often not in senior positions than natives in their workplaces. As such, they were more prone to losing their jobs or to reduced working hours leading to financial insecurity and psychological ill health. Such vulnerability was more profound among migrants during COVID-19 when specific workplaces had to cut down on staff or close [31], and the African migrants in our study were no exception as it contributed to their reduced ability to purchase basic necessities and send remittances to the relatives back home. Although the Government provided an emergency financial support to offset the financial burden, access to such support was limited, especially for African migrants on temporal visas. A similar finding was reported in a survey among international students in Australia, the largest group of long-term temporary visa holders [53].

Despite the negative COVID-19–related psychological impact, COVID-19 lockdowns also brought some positives that helped migrants to cope with the untoward effects of COVID-19. Some of these positives were the opportunity to bond with family, given the fact that people often struggle with a poorer work-life balance leading to little time for family and to slow down, reflect, and be appreciative of life. The findings of the study aligns with previous studies in which adults reported enjoying spending more time with family and developing better relationships due to the extended time together during the COVID-19 lockdown [54, 55]. Such family time has been shown to help to manage everyday challenges and to better understand each other and their feelings during the lockdown. Also, migrants were able to access their superannuation as a way to counter the huge financial burden on individuals and their families. It was observed that migrants received financial and emotional support to go through the challenges of COVID-19 from the Government and community. Even though many benefited from government effort such as access to their superannuation to offset their financial burden, some had difficulty accessing these services. Lack of digital literacy and being on temporal visa were identified as key bottlenecks to accessing COVID-19–related services, and key barriers to accessing services among migrant populations [9]. Community support was a significant source of cultural safety and belonging, and it is in line with the Afrocentric paradigm, which fosters collectivism rather than individualistic approaches. Social support from communities and religious organisations were identified as a protective factor in promoting resilience, hope, and wellbeing during difficult times [56].

In alignment with migrant studies [38, 57], religiosity was identified as a key coping strategy to the COVID-19–related socio-psychological problems they experienced. Contrary to Western societies, religion, and faith are central to how Africans make sense of, and find solace from adverse events they are experiencing [57]. Participants in our study indulged in self-care practices such as using home remedies, Yoga, and media as coping mechanisms to deal with the COVID-19–related stressors. Our finding is in line with results from studies on coping mechanisms employed by migrants during the COVID-19 outbreak [58, 59]. The use of home remedies among African migrants as it has been reported in a previous study among African women refugees in Australia [60], suggest that in time of adversity, African migrants seek solutions from therapies that aligns with their culture. We observed that participant use of social media to socialised with friends and family. However, while the news media was used to keep abreast with news to make informed decision, others stopped listening to the news as it was considered as a trigger of their COVID-19–related stressors. Our finding suggests that different dimensions of the media can serve as a protective factor or expose migrants to COVID-19–related stressors.

### Implications for Health Policy

The findings of the study highlight the socio-psychological toll of COVID-19 public health response activities on African migrant in New South Wales. Policies and programs targeting this subgroup of migrants in Australia should strengthen individual and family resilience and should include culturally responsive and family-based models of mental health care that acknowledge collective experiences and harness community resources to address stressors due to COVID-19 and other future outbreaks. A report by Red Cross Australia suggest that migrant exhibited high levels of resilience because they have gone through migration and settlement barriers[61]. Therefore, community-based interventions focusing on existing strength are likely more effective than vulnerability-centred approaches. A case in point is successfully implementing the culturally adapted racially and culturally minoritised mindfulness program delivered to Arabic and Bangla-speaking communities in Sydney [62].

### Study Limitations and Strength and Future Directions

Readers need to consider the following study limitations when interpreting our findings. First, our study may not be representative of all African migrants in New South Wales since our sample was small, and we used a convenient sampling procedure. However, the fact that we recruited from regional and urban NSW allows us to report African migrants’ views from the whole of NSW. There is a potential for recall bias as we relied on self-reports. Given that this study used a semi-structured qualitative approach to collect data, we sought to hear participants ‘interpretations’ of how they navigated the COVID-19 experience. The reflections during and after this time remain the participants’ own. The research supporting the discussion section is our analysis to the dominant themes in the participant data, which are consistent with a significant number of international studies on minority groups’ experiences during the pandemic [9, 28, 50, 63]. Notwithstanding these limitations, our study is the first to deeply explore the impact of COVID-19 on African migrant wellbeing, their coping strategies, and views regarding COVID-19 vaccine rollout in urban and regional New South Wales.

## Conclusion

The COVID-19 response effort disrupted the collectivist culture of African migrants and had adverse socioeconomic impacts that affected their health and wellbeing. Such impacts highlight an exacerbation of pre-existing inequities. To ensure that African migrant COVID-19–related health and wellbeing needs are met, the African migrant community must be actively involved in conceptualising, designing, and implementing interventions to address the stressors of COVID-19 and future outbreaks. The use and empowerment of community leaders, faith leaders, and health practitioners from the communities is critical. It has been shown that players serve as points of authority that assist in eliminating language and cultural barriers, facilitate cross-cultural understanding and bridge sociocultural gaps, and develop trust and a therapeutic relationship between migrant communities and the health system. Establishing an African migrant community advisory group composed of community leaders, faith leaders, and health practitioners could serve as a strong community consultation and engagement model that can be used at the beginning of future outbreaks.

## Data Availability

The data are not publicly available because of the sensitive nature of its content and concerns surrounding privacy and confidentiality of research participants. The de-identified transcripts of the interviews supporting the findings of this study are available on request from the corresponding author.
